# Systemic Design Approach to a Real-Time Healthcare Monitoring System: Reducing Unplanned Hospital Readmissions †

**DOI:** 10.3390/s18082531

**Published:** 2018-08-02

**Authors:** Faisal Alkhaldi, Ali Alouani

**Affiliations:** Department of Electrical and Computer Engineering, Tennessee Technological University, Cookeville, TN 38501, USA; AALOUANI@tntech.edu

**Keywords:** automaton, discrete-event systems, e-healthcare, hospital readmissions, information and knowledge management system, patient monitoring, real-time

## Abstract

Following hospital discharge, millions of patients continue to recover outside formal healthcare organizations (HCOs) in designated transitional care periods (TCPs). Unplanned hospital readmissions of patients during TCPs adversely affects the quality and cost of care. In order to reduce the rates of unplanned hospital readmissions, we propose a real-time patient-centric system, built around applications, to assist discharged patients in remaining at home or in the workplace while being supported by care providers. Discrete-event system modeling techniques and supervisory control theory play fundamental roles in the system’s design. Simulation results and analysis show that the proposed system can be effective in documenting a patient’s condition and health-related behaviors. Most importantly, the system tackles the problem of unplanned hospital readmissions by supporting discharged patients at a lower cost via home/workplace monitoring without sacrificing the quality of care.

## 1. Introduction

The quality of care (QOC) and cost of care (COC) of healthcare services provided by healthcare organizations (HCOs) has become a front-and-center issue in the United States [1,2]. The general goal of this study is to reduce the COC while improving or maintaining the QOC. The QOC and COC are affected by the management of processes of care (POC) [3]. Through POC, healthcare services, including patient support, assessment, diagnosis, treatment, etc. are delivered to patients [4]. The POC have a direct impact on the following seven major system outcomes inferred from [3,5,6]: (1) timeliness (e.g., patient waiting time); (2) degree of utilization of healthcare services (e.g., overutilization, underutilization); (3) effectiveness of interventions and care provisions (e.g., health improvements, pain containment/management, etc.); (4) (avoidance/occurrence of clinical) adverse events; (5) patients’ satisfaction/experience; (6) mortality; and (7) unplanned hospital readmissions.

For a real-time operational approach, improvements in COC and QOC require measurable components to be *acted upon*, for example, by (reducing) rates of unplanned hospital readmissions related to targeted outcomes, e.g., unplanned hospital readmissions. Attempts to alter these components subsequently take place through interventions taken on certain relevant system state variables. This paper, which is an extension of work that originally appeared in reference [7], takes this approach, while the focus is on reducing unplanned hospital readmissions via real-time support of discharged patients using a proposed monitoring system. This is expected to bring sustainable improvements to QOC and a reduction of COC [6].

The annual cost of hospital readmissions in the United States is around $26 billion [8]. Crucial interventions for reducing readmission rates include transitional care, defined as “a collection of services aimed at ensuring optimal communication and coordination of services to provide continuity of” care [9], effective post-discharge support, sustainable discharge, and extended care [10]. The referenced studies suggest that a post-discharge support program, designed to enhance patient engagement with healthcare organizations and promote a smooth transition from hospital to home, can decrease the number of readmissions. Reference [9] suggests that post-discharge support solutions should be tailored to each patient and include (1) alertness to symptoms of deteriorating conditions and (2) timely information flow between patients and care providers, using electronic means for monitoring conditions and proactive planning. Current monitoring and follow-up practices consist mainly of either calling patients during the post-discharge period or home visits [9,11]. These practices are neither efficient, nor effective [7]. Another solution is to extend patient hospital stays, but this is costly, not always necessary, and could delay other patients being served. An efficient and effective solution with social and economic benefits is needed.

Various proposed systems to monitor patients that could theoretically reduce readmission rates are found in the existing literature [12,13,14,15,16,17,18,19,20]. These systems, however, lack the automation needed to identify at-risk patients in a timely fashion. More details on their individual limitations can be found in reference [7]. However, in more recent advances, the authors in references [8,21,22] proposed to identify patients with a high risk of readmission using (at-rest) clinical and nonclinical data. However, they lack real-time monitoring (i.e., information flow between patients and care providers) during the post-discharge support period, which is vital in providing timely support to patients post-discharge. Finally, as observed by reference [23], only a small number of pertinent papers have been guided by a theoretical framework. 

As discussed in reference [7], our proposed model-based solution requires two essential elements: (1) *a patient-centric approach* [24] for personalized intervention and (2) *a means of communication*, to allow real-time, remote patient-provider interaction, which would act like an offsite “nurse-call” button.

Regarding the *means of communication*, advances in mobile health and medical systems can be found in references [23,25,26,27,28]. These systems have drawbacks: (1) they lack automated processes to identify patients in need of immediate help or intervention; (2) they are commonly activity- or symptom-specific; (3) most are unable to communicate in real-time, and/or (4) there is no guarantee that care providers review each patient and assess his/her condition in a timely manner. 

In the present work, we propose a systemic approach, supported by a control-theoretical framework, that overcomes current limitations in the existing literature. A novel element of our research is the treatment of post-discharge patients and the involved healthcare organization as two interconnected subsystems, together forming a discrete-event dynamic system (DES). This approach provides an important basis for the contributions of this paper: (1) the design of a patient-centric real-time system built around applications (or apps) to facilitate post-discharge patient support in a transparent and effective way; (2) the establishment of an automated process to identify patients at risk for readmission; (3) the inclusion of a higher authority in the process of patient support to automate supervisory functions; and (4) the design of configurable reporters, such that care providers can independently maintain data customized to their patients. This enables the system to account for the uniqueness of each patient and enables the provision of information to an HCO simultaneously from multiple patients.

This paper is organized as follows: Section 2 describes the proposed system and discusses the model structure. Section 3 contains the system model and design development. A discussion, an example, and simulation results are presented in Section 4. Section 5 discusses the conceptual design of the system and a basic plan of implementation; and Section 6 concludes the paper. 

## 2. System Description and Model Structure

Although it can be used in many other healthcare-related applications, we discuss our real-time, patient-centric monitoring system, denoted as *electronic companion care* (eCC) system, in the context of the TCPs. Reference [9] discusses two TCPs or transitional care (post-discharge support) time periods, 4 weeks and 1–3 months, under the models developed by Coleman and Naylor, respectively. The latter is used more often for high-risk, older adults. The main aim of the proposed system is to watch for possible post-discharge complications (which will be treated as system state variables) during this transition period of patient vulnerability, to provide support, and to avoid unnecessary readmissions or suggest readmissions as necessary. As a motivating example, suppose a patient has been admitted for surgery. The patient is hospitalized for 2 days and then discharged. Of course, there is always the possibility of post-discharge complications; this is the origin of high rates of unplanned readmission [6]. Assume the patient is classified as requiring 4-week discharge support. Under the traditional approach [9], the hospital staff would check on the patient within the first 1 or 2 days, hoping that no complications arise in the remainder of the 4-week period. If the patient exhibits symptoms after the follow-up, then there are two possible undesirable cases: (1) the patient may seek readmission, although the symptoms could be managed from home (previous research indicates this is a common scenario [9,29]); or (2) the patient ignores the symptoms, does not seek help, and acquires more complications, possibly leading to readmission for a more serious condition. Hence, HCOs need a real-time, more automated, efficient, and effective way of handling this transition period for each patient uniquely. The eCC system aims to fulfill this need.

To this end, the proposed *patient-centric approach* has two main characteristics: (1) the focus centers on and is *customized* to the patient at all times; and (2) it facilitates a patient’s engagement in his/her own care. These two essential characteristics impose two interrelated requirements: (1) each patient must be treated as a unique entity and (2) the system must be designed at the patient level. With this in mind, we can continue as follows.

**Figure 1:**
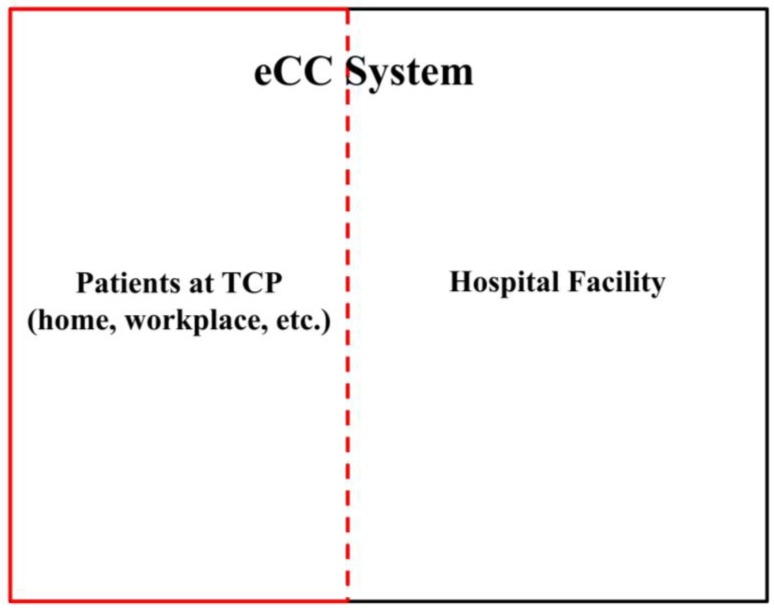
Aggregate view of an electronic companion care (eCC) system. TCP: transitional care period.

Patients in TCPs are monitored by the involved hospitals or any other HCO. It is known that a system, e.g., a hospital system, can be distinguished from its environment by the limits of control exercised by its components. These limits define the system boundary [30]. Therefore, the eCC system “extends” the hospital system boundary to include patients in TCPs. This inclusion is coarsely shown in Figure 1.

In a top-down approach, we first identified the system at its highest level (Figure 1). Looking deeper into the system, the subsystems can be decomposed as follows.

### 2.1. The Patient’s Subsystem—The Initiator

This subsystem generates health-related events based on patients’ symptoms and conditions. Health-related activities, symptoms, and conditions can be represented by a certain number of *finite*, *discrete states*. Recalling the example discussed in reference [7], in terms of bleeding, a patient might be in one of two states: *bleeding* or *idle* (no bleeding observed). As different symptoms and/or conditions develop, the patient transitions between different states. We designate the transitions between these states as *events.* In this manner, patient behavior can be described as a *finite state automaton*, comprising sequences of events that lead to states experienced by the patient as his/her health and health-related activities evolve. To model the system, we used a branch of system theory [31], termed *sequential machines and automata* (e.g., finite automata) which manages this type of system behavior. A *finite automaton* can be informally defined as a machine that can be in one or more states among a set of possibilities depending on its previous state and the most recent event. One describes the dynamics of an automaton using formal language over an alphabet. In our research, we modeled a single patient as a finite-state machine, denoted by *patient automaton* $PA_{io}$, where the subscript o means outside healthcare organizations (walls), and i∊ℕ, i=1,2 …,k, where k is chosen based on the HCO patient discharge data or bed capacity to represent the maximum possible number of discharged patients in TCPs that can be monitored simultaneously. 

### 2.2. The Care Provider Subsystem—The Inquirer

The Care Provider Subsystem comprises three concurrent components: (1) the nursing station; (2) a higher healthcare professional authority, e.g., hospitalist; and (3) a configurable reporter in the communication link between the patient (initiator, $PA_{io}$) and care provider (denoted by $M_{i}$). The main advantage of this monitoring system is that it facilitates patient transitions to all possible states of symptoms, health conditions, and health-related activities, yet calls the attention of care providers to only those patients currently at risk for readmission. This requires the care provider subsystem to recognize key events (i.e., patient warning signs) as they occur for each patient in a TCP. For event recognition, we designed the inquirer subsystem in an event-based manner, coupled with the patient subsystem; in other words, certain patient events trigger corresponding care provider transitions. The first step towards meeting this requirement is modeling the inquirer subsystem as a *finite state machine*. The reporters allow only the events that represent certain dynamics (behaviors) of interest (i.e., key symptoms or conditions) to pass from the patient to the care providers. The reporters must be designed in a manner that (1) captures the uniqueness of each patient; and (2) can be adjusted by the care provider to monitor a list of symptoms on the basis of patient-dependent health developments. We used *natural projection* [31] to model such reporters.

**Figure 2:**
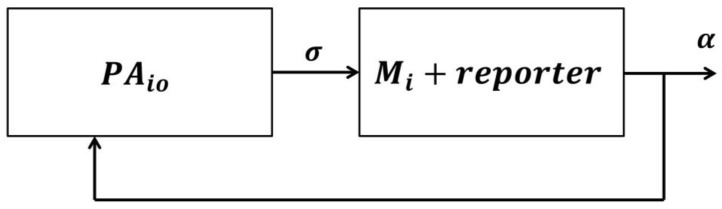
Block diagram of the eCC initiator, $\mathrm{patient}\;\mathrm{automaton}\;PA_{io}$, and care provider subsystem: $M_{i}$ with the reporter.

After adding these details to the system in Figure 1, the representation can be illustrated as shown in Figure 2.

The subsystem $PA_{io}$ in Figure 2 represents patients in TCPs. We represent all possible events by symbols (e.g., σ) that are elements in an alphabet (Σ) of the patient automaton, σ∊Σ. Events represent patient behavior outside healthcare organizations, such as the patient transitioning to a pain state, bleeding, etc. They are delivered through configurable reporters C to the prescribed care provider (automata, $M_{i}$) which transitions over to its finite event set Τ, α∊T, as a response to these received events from the patient automaton. In this patient-centric approach, the aim is to intervene in a timely manner whenever patients need assistance, e.g., when a patient transitions to an unwanted health condition (or symptom) that indicates a higher risk of potential unplanned readmission. For this purpose, the two subsystems, $PA_{io}$ and $M_{i},$ in Figure 2 are coupled via shared events.

**Definition** **1.** *(Coupling events): Let Σ and Τ symbolize the transition labels of the patient automata and care provider, respectively. In this context, we can define the system with (1) and (2). We denote the elements in set Ε as the coupling events*

(1) Τ ∩ Σ ≠ ∅,

(2) Ε={σ | σ∊ Σ & σ∊Τ }.

**Figure 3:**
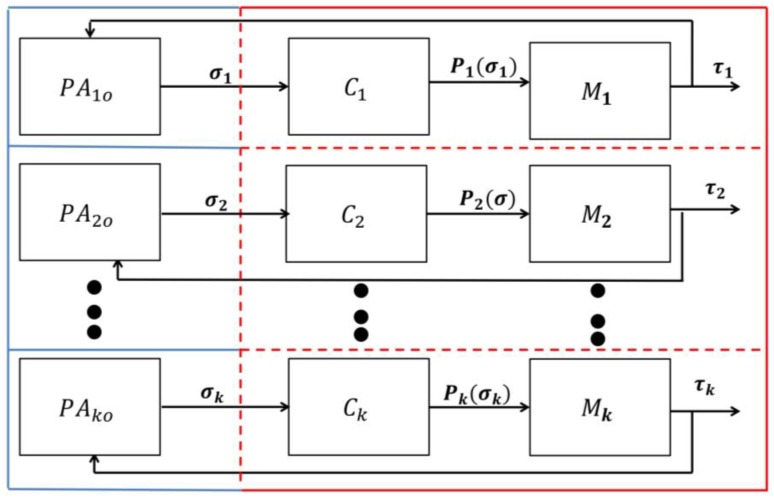
The components of an inquirer subsystem (within the red box) and initiator subsystem (within the blue box). The components $PA_{io}$, $C_{i}$ and $M_{i}$ form a monitoring agent (*i*) with a reporter.

In other words, the two finite alphabet sets, Σ and Τ, must have elements (events) in common, for coupling purposes. This coupling approach can mimic intelligent behavior in hospitals, in particular, the behavior of delivering timely “care” to patients based on their needs. This was implemented in eCC as follows. First, we incorporated all possible symptoms and condition transitions into both the patient automata models $\left(PA_{io}\right)$ as well as into the care provider model. Hence, we effectively implemented the “nurse-call” button behavior commonly used in hospitals. To allow for transparent processes, we also incorporated the events of symptom investigations in both models; in this way, patients can be fully aware that their concerns are being handled and receive attention by care providers as if they were still in the hospital. As can be seen in Figure 3, a care-provider-automata with a reporter is assigned for every individual patient automaton currently in a TCP (analogous to how every patient in a hospital has his/her own nurse-call button). Upon completion of the TCP, the patient automaton is disconnected and can be replaced by another (potential) discharged patient. The patient’s identifier could be his/her phone number (or some other suitable unique identifier).

With these incorporations, now, when a patient enters a TCP, for efficiency, effectiveness as well as patient uniqueness realization, the involved hospital can configure the reporter to select personalized key symptoms and conditions for exposure as they occur, such that only the corresponding key events are allowed to go through. Once any of the allowed/observable events are received by the care provider automaton, it will make it transition to a state of symptom investigation, as will be seen later. The ongoing process of investigation will communicate back to the corresponding patient to assure that reported concerns are being addressed and he/she is currently under “care”.

In this way, the eCC system works in accordance with the basic principles of a discrete event system (DES) with shared events [31], which can be illustrated as follows. A patient automaton $PA_{io}$ and a care provider station automaton $\left(M_{i}\right)$ (i∊ℕ, i = 1, 2, …, k, where k is equal to the number of care provider automata involved in monitoring patients, one for each patient in a TCP) are placed in a closed loop (Figure 3), assuming they are connected wirelessly. The components $PA_{io}$ and $M_{i}$ are together called the *monitoring agent i*. If a patient automaton transitions over an event (σ), then a transition can be signaled, as permitted by the reporter ($C_{i}$), to $M_{j}$ which itself will generate the corresponding event (σ) if (i) σ∊ Τ and (ii) σ can be catenated after $\tau ∊{Τ}^{*}$ (i.e., if it is “state enabled” or if the execution is “physically possible”), where the set ${Τ}^{*}$ includes all finite strings over the alphabet Τ (Figure 3). The detailed model of each component in the eCC system is discussed in the next section.

It is worth noting that the feedback line in Figure 3 includes a point in the loop for human intervention. Care provider intervention could include contacting the patient to schedule a visit in the patient’s home, coordinating patients to go to the nearest healthcare organization, asking patients to return to the hospital for readmission (when medically necessary), or simply providing instructions to manage the patient’s condition and symptoms [6].

## 3. The System Model and Design Development

As discussed earlier, every patient is considered to be a finite state automaton. It will be shown that the state space of this *patient automaton* consists of a hierarchal and concurrent structure, thus we denote its model as a *Structured Patient Automaton (SPA).* This type of structure is also observed in the care-provider subsystem automata. Therefore, the *state tree structure* (STS) modeling technique, developed by Ma and Wonham (2005) [32], was used to model the eCC system. It provides supporting tools to such structured automata. The STS’s state space features modules, namely *holons*, by which the *local* behavior of the automaton can be modeled. Through the holons’ interactions, *global* behavior can be ensued/represented. The STS technique handles the structure complexity through introducing state aggregation-decomposition and layering to the model. These are represented in the technique through compact graphical representation by the notion of superstates and their components, the local states, which can be simple states.

As an STS DES, a compact mathematical representation of the eCC STS model can be given by a 6-tuple G, as follows:

(3) $$G=\left(ST,\;ℋ,\;\Sigma ,\;\Delta ,\;ST_{0},\;ST_{m}\right),$$

where $ST≔\left(X,\;x_{0},\;T,ℰ\right)$ is a 4-tuple structured state space, in which X is a finite structured state set and $x_{0}∊X$ is the root state. T:X→{AND, OR, simple} is the type function. ℰ:X→ƿ(X) is the expansion function [32], where ƿ(X) is the power set of X. $ℋ≔\{H^{a}|T\left(a\right)=or\;\&\;H^{a}=\left(X^{a},\;\Sigma^{a},\;\delta^{a},\;X_{0}^{a},\;X_{m}^{a}\right)\}$ is a set of matching holons assigned to all of the OR (the semantic of OR is the disjoint union of states) superstates of ST. Σ is the event set including all of the events appearing in ℋ. $\delta^{a}$ is the transition structure, $\delta^{a}:X\times \Sigma \to X$, of the holon, $H^{a}$. Δ is the global transition function. $ST_{0}∊ST\left(ST\right)$ is the *initial state tree*. $ST_{m}\subseteq ST\left(ST\right)$ is the *marker (or final) state tree set*.

The $PA_{io}$ and $M_{i}$ components belong to the monitoring agent (i), denoted $G_{i}$, (i∊I, I an index set). We viewed G as being comprised of a group of monitoring agents, $G_{i}$, acting concurrently and independently (Figure 3). The $G_{i}$ agents are defined over pairwise disjoint alphabets $\left(\Sigma_{i}\right)$ such that $\Sigma =\dot{\cup }\{\Sigma_{i}|i∊I\}$. This permits the following equation [33]:

(4) $$G=|{|}_{i=1}^{m}\;G_{i},$$

where ‖ denotes the concurrent composition of the monitoring agents. In other words, G is the *shuffle product* of $G_{i}$ [31,33]. In this way, the interconnected components of each monitoring agent $(G_{i}$) can be modeled and designed independently as an STS DES, and then eCC can be obtained mechanically by combining the monitoring agents $\left(G_{i}\right)$ in a way to operate concurrently. With this, $G_{i}$ can be now given by

(5) $$G_{i}=\left(ST_{i},\;{ℋ}_{i},\;\Sigma_{i},\;\Delta_{i},\;ST_{0_{i}},\;ST_{m_{i}}\right),$$

with the 6-tuple, as discussed earlier. For each of the monitoring agents $\left(G_{i}\right)$ in eCC, we need to develop the model of its constituent components, $PA_{io}$ and $M_{i}$. In this section, we develop the patient automaton $PA_{io}$ model first. This is followed by modeling the care provider automata, $M_{i}$, and the configurable reporter, $C_{i}$.

### 3.1. The Universal Patient Model

The two requirements of the *patient-centric approach* discussed in the last section contributed to the introduction of the concept of the *universal patient model* (UPM) [7]. The universality can be attributed to two main features: (1) it is generic and can be applied to any patient, independent of his/her medical conditions and attributes while maintaining his/her uniqueness; and (2) it represents the dynamical behavior of the patient both in and outside healthcare organizations. The UPM is represented by an SPA.

**Figure 4:**
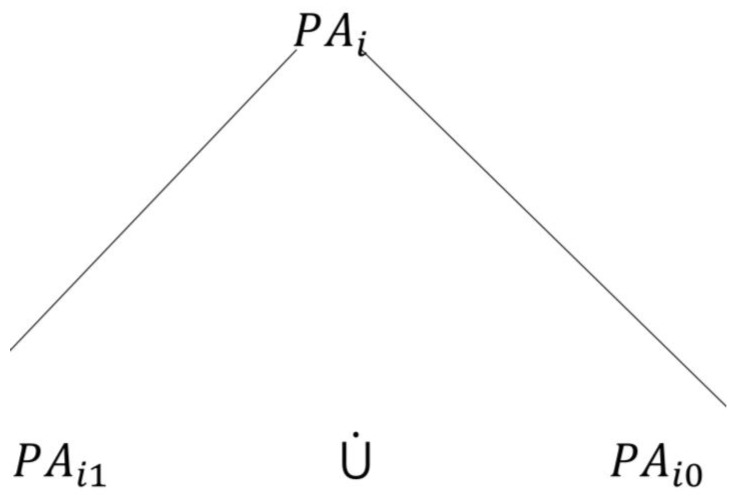
The global states of a $PA_{i}$.

Starting at the highest level, the UPM has a *root* state denoted as $PA_{i}$. This root state can be expanded by two *global* states (Figure 4), denoted as $PA_{i1}$ and $PA_{io}$, where the patient is in the healthcare organization and the patient is outside the healthcare organization, respectively, as follows:

(6) $$ℰ\left(PA_{i}\right)=\left\{PA_{i1},\;PA_{i0}\right\}.$$

For the purpose of this work, this paper only discusses the part of UPM when patients are outside healthcare organizations. In reference [4], we introduce the other portion of UPM for patients inside healthcare organizations, for a different use.

The SPA models the patient dynamics on a *logical level*, as discussed in the example in reference [7]. As given by reference (5), we first develop the finite state space $\left(ST_{i}\right)$ applicable to all patient automata, $PA_{io}$:

(7) $$ST_{i}≔\left(X,\;PA_{io},\;T,ℰ\right),$$

where, for our purpose, $PA_{0i}∊X$ is now made the root state, instead of $PA_{i}$, which allows $X={ℰ}^{*}\left(PA_{io}\right)$. The patient automata have identical state spaces, according to the (child-)state tree topology that decomposes $PA_{io}$, but each patient automaton $PA_{io}$ evolves uniquely in accordance with his/her unique symptoms and medical conditions by executing the corresponding transitions. We discuss this more later. The global/root AND-state $PA_{io}$, to which the patient automaton transitions when the patient enters a TCP, can be expanded by all possible symptoms that a patient may experience using the expansion function (ℰ) [32], as follows:

(8) $$ℰ\left(PA_{io}\right)=\left\{s_{j}\right\},\;j=1,\ldots ,38,$$

where $s_{1}$ is the pain superstate, $s_{2}$ is the fatigue superstate, $s_{3}$ is the headache superstate, $s_{4}$ is the infection superstate, $s_{5}$ is the swelling superstate, $s_{6}$ is the fever superstate, and the rest of the superstates are developed in a discussion found in Appendix A. Each symptom superstate is expanded by its components as follows:

(9) $$ℰ\left(s_{j}\right)=\left\{s_{jo},\;s_{j1},\;s_{j2},\;s_{j3},\;s_{j4}\right\},$$

where the *simple state* $s_{jo}$ means the patient has no evidence of the particular symptom, (e.g., fever), $s_{j1}$ means the patient is experiencing the symptom, $s_{j2}$ means the symptom is under investigation by the care provider, $s_{j3}$ means that the concern about the symptom has been addressed and a care plan is waiting for the approval of a qualified healthcare professional (e.g., hospitalist), and $s_{j4}$ means that to the intervention to avoid the symptom has been approved and patient is still experiencing the symptom. There is a transition from $s_{j4}$ back to $s_{jo}$ when the symptom is relieved. This is addressed more when we discuss the dynamics of the system later in this work.

**Figure 5:**
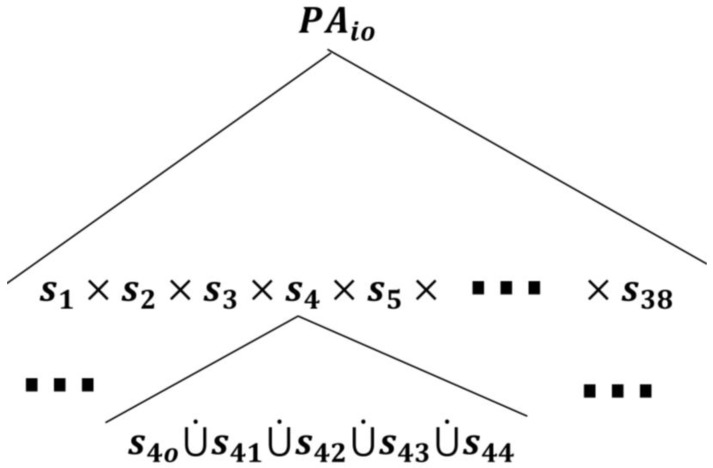
Represents the state space topology of a patient automaton $PA_{io}$. Other superstates $\left(s_{j}\right)$ can be expanded as shown for $S_{4}$. We limited the expansion to the components of $S_{4}$ due to space limitations.

With these developments, the expansion of (7) and (8) can be shown graphically, as shown in Figure 5. The semantics of $PA_{io}$ is the Cartesian product of all patient symptoms $\left(s_{j}\right)$. Basically, the $PA_{io}$ must have a local (or simple) state in each and every component $\left(s_{j}\right)$ simultaneously, whereas, the semantics of $s_{j}$ is the *exclusive-or* (XOR), i.e., the disjoint union of states. In other words, the system must be at *one state* of $s_{j}$.

As shown in Figure 5, each patient in a TCP has 38 concurrent superstates representing (almost) all possible symptoms, each of which has 5 possible *local/simple* states, as discussed earlier. The model is flexible, as required, so that additional health-related activities or a missing symptom can be readily added. Equally, an existing one can be removed, if desired. For example, if medication compliance needs to be closely monitored during the first two weeks after discharge, then it can be added where transitions under its *holon* can be used to exchange information with the care provider about the targeted health-related activity. Moreover, any symptom can be further disaggregated to provide more detailed information. For example, the pain superstate can be further disaggregated into the possible types of pain that may occur, for example, there are superstates for chest pain, muscle pain, back pain, abdominal pain, joint pain, etc. One superstate for each can be established.

After developing the generic state space topology for patients outside HCOs, the rest of the UPM was dedicated to the dynamics and certain aspects of the design. According to Figure 5, the $PA_{io}$ holons can be given by

(10) $${ℋ}^{PA_{io}}≔\{H^{s_{j}}|T_{i}\left(s_{j}\right)=or\;\&\;H^{s_{j}}=\left(X^{s_{j}},\;\Sigma^{s_{j}},\;\delta^{s_{j}},\;X_{0}^{s_{j}},\;X_{m}^{s_{j}}\right)\;\&\;j=1,\ldots ,\;38\},$$

where $\Sigma^{PA_{io}}=\dot{\cup }\{\Sigma^{s_{j}}|\;j=1,\ldots ,\;38\}$ is the event set including all of the events labeling transitions between *local* states in ${ℋ}_{i}^{PA_{io}}$. The events can be patient-enabling or care-provider-enabling; thus, $\Sigma^{s_{j}}$ can be partitioned by $\Sigma^{s_{j}}=\Sigma_{pt_{j}}\;\dot{\cup }\;\Sigma_{cp_{j}}$. All events are disabled in $H^{s_{j}}$ until they are enabled for execution by a patient for $\Sigma_{pt_{j}}$ and by a care provider for $\Sigma_{cp_{j}}$ with the condition $\Sigma_{pt_{j}}\;\cap \;\Sigma_{cp_{j}}=\emptyset$, where $\Sigma_{pt_{j}}$ is the set of patient-enabling events and $\Sigma_{cp_{j}}$ is the set of care-provider-enabling events. We further partitioned the care-provider-enabling events into two disjoint subsets, $\Sigma_{cp_{j}}=\Sigma_{ncp_{j}}\;\dot{\cup }\;\Sigma_{hcp_{j}}$: (1) events only enabled by the nursing station, $\Sigma_{ncp_{j}}$; and (2) events only enabled by a higher healthcare professional authority, e.g., a hospitalist, $\Sigma_{hcp_{j}}$. We also require that $\Sigma_{ncp_{j}}\;\cap \;\Sigma_{hcp_{j}}=\emptyset$. Notice that events may be enabled by an external agent (i.e., by a human or a machine). To distinguish the different types of events on the eCC STS transitional models, we labeled (1) $\Sigma_{pt_{j}}$ with odd numbers; (2) $\Sigma_{ncp_{j}}$ with even numbers; and (3) $\Sigma_{hcp_{j}}$ with letters and even numbers in the subscripts. To avoid generating the numbers of events manually, which could be cumbersome given the potentially large number of concurrent patients in TCPs [34] and *holons* involved, we show, in Appendix B, a systematic way to create the number of each event which appears in the holons of all patient automata $\left(PA_{io}\right)$ in the eCC.

**Figure 6:**
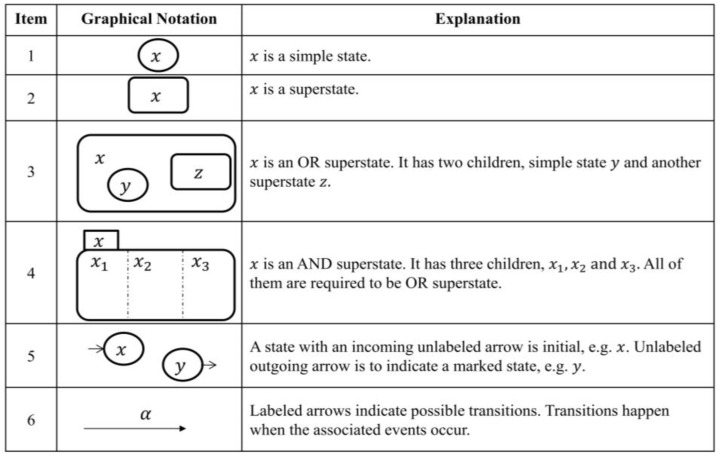
The main graphical notation of the transitional model [32].

The STS model represents the dynamical model, referred to as the *transitional model*, graphically. It is basically a structured model for automata. A mathematical discussion of transition functions of the STS model is detailed in reference [32]. The transitional model was built using holons which together make up the associated $PA_{io}$ behaviors. This is possible due to the capability of a holonic approach to model an ordered system (as opposed to chaotic) of related “processes”, where each “process” itself is a holon. A holon can be informally defined as an autonomous entity which itself consists of a collection of cooperative group of holons that work together for a certain overall goal. A fundamental component needed to construct an STS model is to establish a state space topology of the system (alternatively called a state tree) which has been developed and presented in Figure 5. The main graphical notations of an STS transitional model are given in Figure 6.

**Figure 7:**
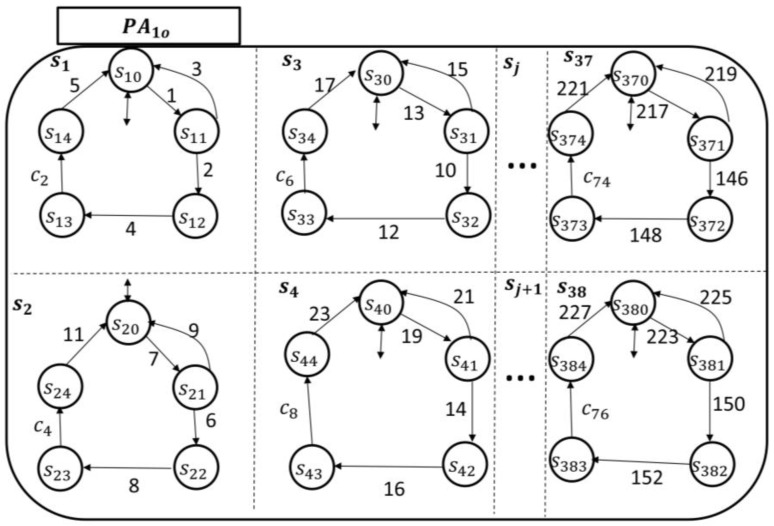
Patient automaton transitional model.

The transitional model was built based on the $PA_{io}$ state space topology and the pertinent dynamics/behavior of patient (automaton $PA_{io}$) outside formal HCOs, as shown in Figure 7. The rest of the patient automata $PA_{io}$ i∊ℕ, i=2,…,k can be built in a similar fashion. Notice, for example, in the holon $s_{1}$ of $PA_{io}$, the events are $\left\{1,3,5\right\}\subset \Sigma_{pt}$. They represent the patient starting to have pain, the pain being relieved, and the pain being relieved with care provider intervention, respectively. The patient cannot have any of these events executed if they are not state-enabled, e.g., if $s_{1}$ is at $s_{12}$, or $s_{13}$. Event 3 is needed because it allows the patient to go back to a normal state (i.e., symptom is relieved). This is mostly required when the corresponding symptom is not one of the monitored ones, thus it will not go through the dynamics of care-provider investigation. As the events $\left\{2,4\right\}\subset \Sigma_{ncp}$, they, therefore, represent the nursing station starting to investigate/develop a care plan for the pain symptoms and the care plan being finalized, respectively. The events {2,4} must not occur in the patient automaton unless they are enabled by the nursing station at the holon $n_{1}$; we discuss this further in the next section. The event $\left\{c_{2}\right\}\subset \Sigma_{hcp}$, it means the proposed care plan has been approved by a qualified health professional. It is enabled by a health professional supervisor at $h_{1}$ (refer to the next section). In a similar manner, these events’ representations can be generalized for all potential symptoms.

### 3.2. Care Provider Automata Model 

**Figure 8:**
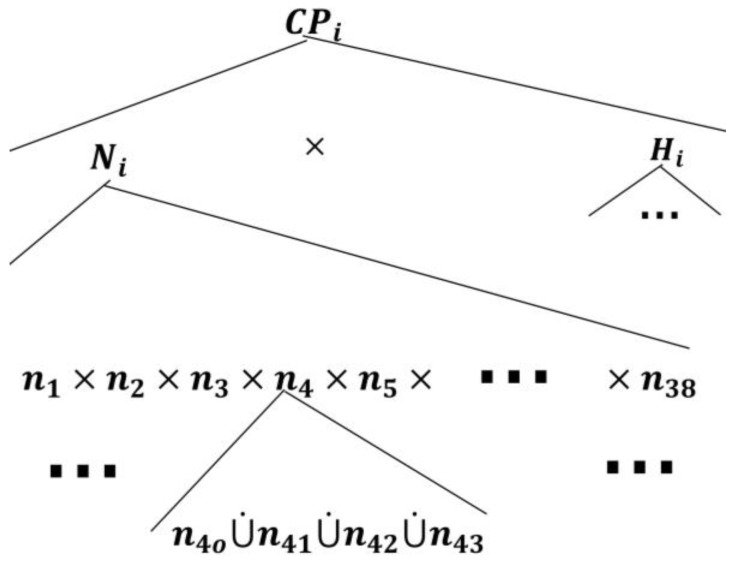
The $M_{i}$ state space topology with the nursing station, $N_{i}$, expanded.

In the care provider, $M_{i}$, automata model, there are two concurrent components as discussed in the *inquirer* section: (1) the nursing station, denoted by $N_{i}$, and (2) a higher healthcare professional authority, e.g., hospitalist, denoted by $H_{i}$ (refer to Figure 8).

The root AND-state $CP_{i}$, to which the care provider automata transitions when connected with a TCP patient automaton, can be expanded by two AND-superstates, namely $N_{i}$ and $H_{i}$, as follows:

(11) $$ℰ\left(CP_{i}\right)=\left\{N_{i},\;H_{i}\right\},\;i=1,\ldots ,k.$$

The component $N_{i}$ is expanded by:

(12) $$ℰ\left(N_{i}\right)=\left\{n_{j}\right\},\;j=1,\ldots ,38,$$

where $n_{1}$ is the pain symptom investigation superstate, $n_{2}$ is the fatigue symptom investigation superstate, $n_{3}$ is the headache symptom investigation superstate, $n_{4}$ is the infection symptom investigation superstate, $s_{5}$ is the swelling symptom investigation superstate, $n_{6}$ is the fever symptom investigation superstate, and the rest of the superstates can be developed based on the discussion provided in Appendix A. The components of each symptom investigation superstate can be given by

(13) $$ℰ\left(n_{j}\right)=\left\{n_{j0},\;n_{j1},\;n_{j2},\;n_{j3},\;n_{j4}\right\},$$

where the *simple state* $n_{j0}$ means the nursing station is not noticing a particular symptom (e.g., fever) reported by the $PA_{io}$ (either no symptom has been reported, or the patient has generated an event corresponding to the occurrence of a symptom, but the reporter has erased it from passing through to the care provider, as will be explained next); $s_{j1}$ means that the nursing station has been informed of the symptom by $PA_{io}$; $s_{j2}$ means that the symptom is under investigation by the nursing station; $s_{j3}$ means that the concern about the symptom has been addressed and the a care plan is waiting for approval by a qualified healthcare professional (e.g., hospitalist); and $s_{j4}$ means that the care plan to avoid the symptom has been approved.

**Figure 9:**
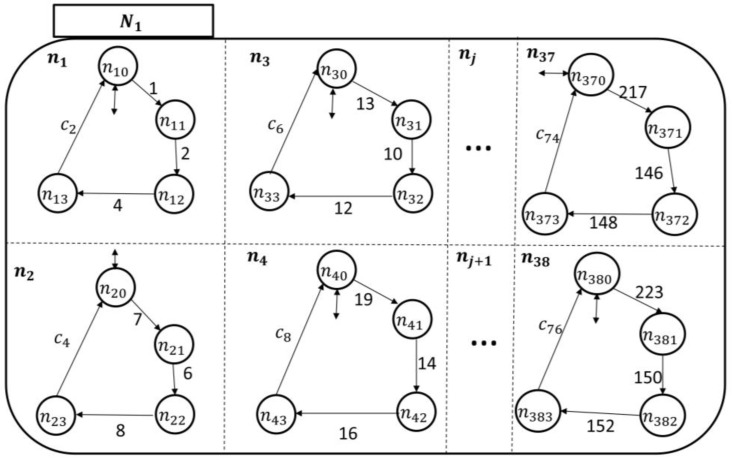
The nursing station automaton transitional model.

Since each $n_{j}$ in $N_{i}$ monitors its $s_{j}$ counterpart in $PA_{io}$, one can follow the discussion presented under the UPM to develop the transitional model of $N_{i}$, as shown in Figure 9. Notice that there is only one patient-enabling event in each holon in $N_{i}$: This is to notify the nursing station about an ongoing symptom/concern at the patient end. The rest of the “processes” in $n_{j}$ are related to the development of care plans and approval, as discussed earlier.

**Figure 10:**
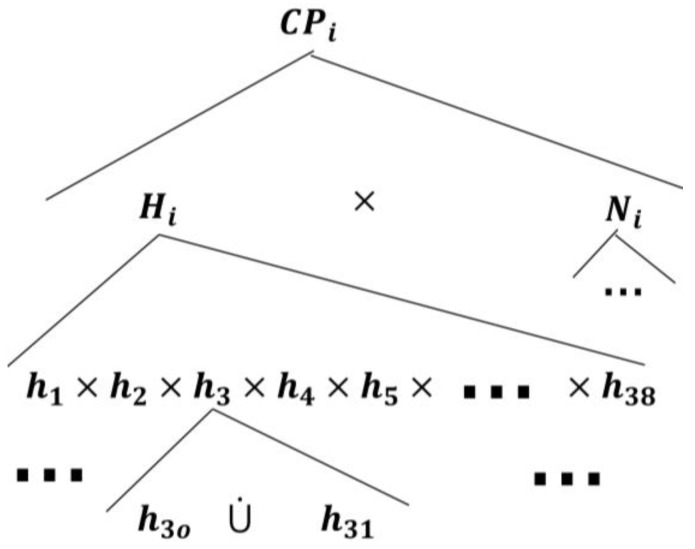
The $M_{i}$ state space topology with the hospitalist, $H_{i}$, expanded.

The state space topology, shown in Figure 10, of the higher health professional authority component $H_{i}$ is expanded by

(14) $$ℰ\left(H_{i}\right)=\left\{h_{j}\right\},\;j=1,\ldots ,38,$$

where $h_{1}$ is the pain symptom care-plan development, review, and approval superstate; $n_{2}$ is the fatigue symptom care-plan development, review, and approval superstate; $n_{3}$ is the headache symptom care-plan development, review, and approval superstate; $n_{4}$ is the infection symptom care-plan development, review, and approval superstate; $s_{5}$ is the swelling symptom care-plan development, review, and approval superstate; and $n_{6}$ is the fever symptom care-plan development, review, and approval superstate. The rest of the superstates can be developed based on the discussion in Appendix A as well. The components of each symptom care-plan review and approval superstate can be given by

(15) $$ℰ\left(h_{j}\right)=\left\{h_{jo},\;h_{j1}\right\},$$

where the *simple state* $h_{jo}$ means that the hospitalist has not received an indication of a particular symptom (e.g., fever) from $PA_{io},$ and $s_{j1}$ means that the hospitalist is reviewing/developing and approving a care-plan for $PA_{io}$ to avoid this particular symptom.

**Figure 11:**
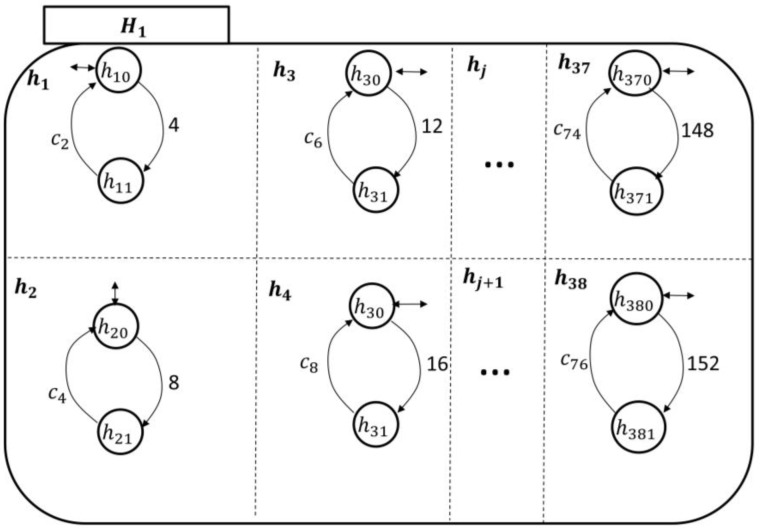
The hospitalist automaton transitional model.

The transitional model of the hospitalist is simpler, as displayed in Figure 11. It shows that the nursing station initiates a report about the patient’s concern, which is thereby communicated to the corresponding $h_{j}$ supervisory. At this point, the hospitalist becomes aware of the case and responds accordingly, as discussed briefly in Section 2. The nursing station may need to call the patient over the phone to further inquire about the symptom which can be included in the electronic report to the hospitalist. The process of sending the electronic report can be linked to the nursing-station enabling event, exiting state $h_{jo}$ to $h_{j1}$ to automate the transitions’ execution.

### 3.3. Configurable Reporter Model

The configurable reporter $\left(C_{i}\right)$ is placed in the channel linking the patient automaton $PA_{io}$ and the care provider automata $N_{i}$ and $H_{i}$. Suppose that the care provider only wants to react to certain key symptoms, not all of the symptoms. Then, only a corresponding subset of the *coupling events* generated by the patient automaton (as he/she transitions between states) is observable by $N_{i}$, i.e., ${Ε}_{o}\subseteq Ε$ or ${Ε}_{o}\subset \Sigma_{pt}$, since a configurable reporter only affects the patient-enabling events. This is the core role of the reporters, $C_{i}$, associated with each monitoring agent $\left(G_{i}\right)$. To mathematically model $C_{i}$, we used the *natural projection* of a formal language [31], as follows

$$P:\Sigma_{pt}\to {Ε}_{o}$$

This is defined as

(16) $$\begin{array}{c} P\left(\epsilon \right)=\epsilon \\ \sigma ∊\Sigma_{pt}\\ P\left(\sigma \right)=\{\begin{array}{c} \sigma \;\mathrm{if}\;\sigma ∊{Ε}_{o}\\ \epsilon \;\mathrm{if}\;\mathrm{otherwise} \end{array} \end{array}$$

where ϵ is an empty sequence (sequence with no symbols). Therefore, the effect of P is effectively to erase the event (σ) that does not belong to ${Ε}_{o}$. These properties are similar to what was previously discussed in reference [31], except that in our application, we do not need the *catenative* property of P. This natural projection (P) can now be called an ${Ε}_{o}$*-symptoms-reporter*.

## 4. Discussion and an Example

In Section 2, it was discussed that there are two main subsystems in the eCC that interact with each other, whereby each patient automaton $PA_{io}$ is linked to $a\;CP_{i}$ care provider through a configurable reporter, $C_{i}$. Each $C_{i}$ reporter channel is associated with a specific $P_{i}$ function that operates over the $\Sigma_{{pt}_{i}}$ alphabet of the patient automaton $PA_{io}$, so that the monitoring process can be uniquely tailored to each patient’s health condition, where efficiency and effectiveness are expected to increase. Certain prescribed event(s), $\sigma ∊{Ε}_{o_{i}}\subset \Sigma_{{pt}_{i}},$ pass through to cause corresponding transitions at the care provider automata end, where ${Ε}_{o_{i}}\subset \Sigma_{cp_{i}}$, ${Ε}_{o_{i}}$ contains *coupling and observable events*, i.e., ${Ε}_{o_{i}}\subseteq E$. Of course, a care provider could choose to be exposed to all of the represented symptoms and conditions in the system, provided that the patient agrees. Therefore, each provider reacts to key symptoms or conditions in accordance not only with the nature of the delivered care, but also with respect to the uniqueness of a patient and his/her preferences. The transition dynamics of the care provider (including both the nursing automaton and hospitalist automaton, as a response to the reported observable symptoms of the corresponding patient) are communicated back to the patient. In this way, the patient is certain that his/her recent health developments are being addressed and is aware of what stage the symptom investigation has reached. Needless to say, this process facilitates transparency, which can improve the QOC, as suggested by the Institute of Medicine in their report, Crossing the Quality Chasm: A New Health System for the 21st Century [35].

The collection of all transitions undertaken by the patient automaton, regardless of visibility to the provider, comprises the dynamic behavior of a patient (see Section 5 for further details). In this manner, the system creates a detailed, time-stamped health-related event record for the patient, which can improve the quality of care and reduce the care service time when a patient is present at a healthcare organization for the following reasons:

(1) Daily health-related events are documented over time. Thus, this eliminates the need for a nurse or physician to ask the patient routine questions and record answers (which may be ineffective due to language barriers, memory lapses, etc.). This may also aid in improving the overall throughput time in healthcare organizations.
(2) Having detailed information available and accessible over a longer period of time enables physicians to improve analysis and diagnostic efforts. Furthermore, since this information can be accessed by a physician ahead of a patient’s visit, more time is available to study the patient’s condition. The physician can spend more time focusing on care provision, rather than collecting information and conducting analyses.

**Figure 12:**
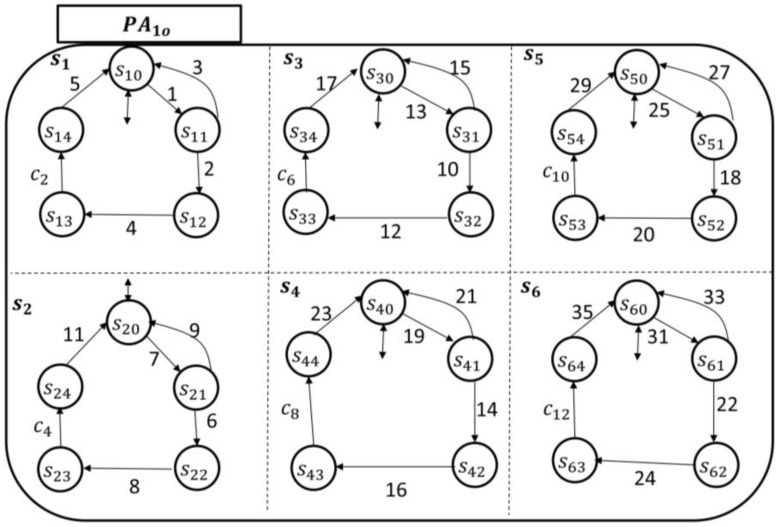
Patient automaton $PA_{1o}$ transitional model.

**Figure 13:**
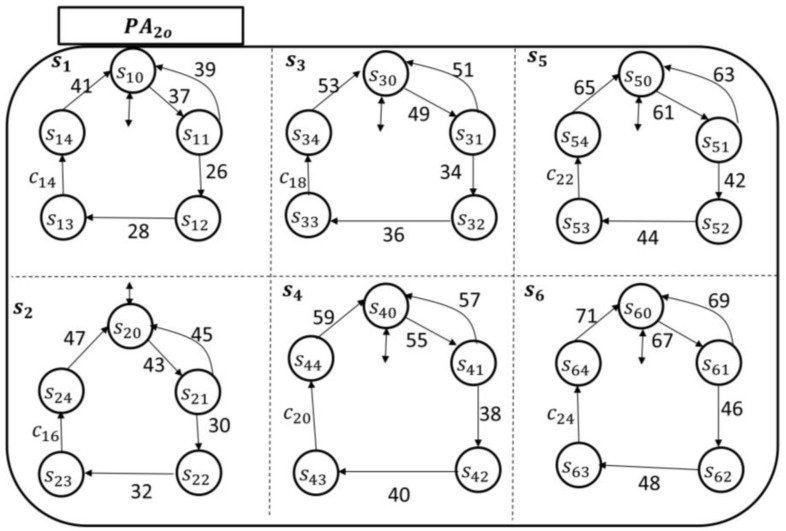
Patient automaton $PA_{1o}$ transitional model.

As an illustrative example of the eCC functionalities, we assumed k=2 (refer to Section 2 for the parameter k), and for simplicity, the discussion is limited to six different symptoms: $s_{1}$ is the pain superstate; $s_{2}$ is the fatigue superstate; $s_{3}$ is the bleeding superstate; $s_{4}$ is the infection superstate; $s_{5}$ is the swelling superstate; and $s_{6}$ is the fever superstate. The transitional model of each $PA_{io}$, where i=1, 2, is shown in Figure 12 and Figure 13, respectively.

The patient-enabling events for each patient automaton are given, respectively, by

(17) $$\Sigma_{{pt}_{1}}=\left\{1,3,5,7,9,11,13,15,17,19,21,23,25,27,29,31,33,35\right\}$$

and

$$\Sigma_{{pt}_{2}}=\left\{37,39,41,43,45,47,49,51,53,55,57,59,61,63,65,67,69,71\right\}.$$

This establishes the initiator’s components. The inquirer’s components are $CP_{1}$ and $CP_{2}$. The enabling-events of the nursing station in $CP_{1}$ and $CP_{2}$ can be detailed, respectively, by

(18) $$\Sigma_{{ncp}_{1}}=\left\{2,4,6,8,10,12,14,16,18,20,22,24\right\}$$

and

(19) $$\Sigma_{{ncp}_{2}}=\left\{26,28,30,32,34,36,38,40,42,44,46,48\right\}.$$

**Figure 14:**
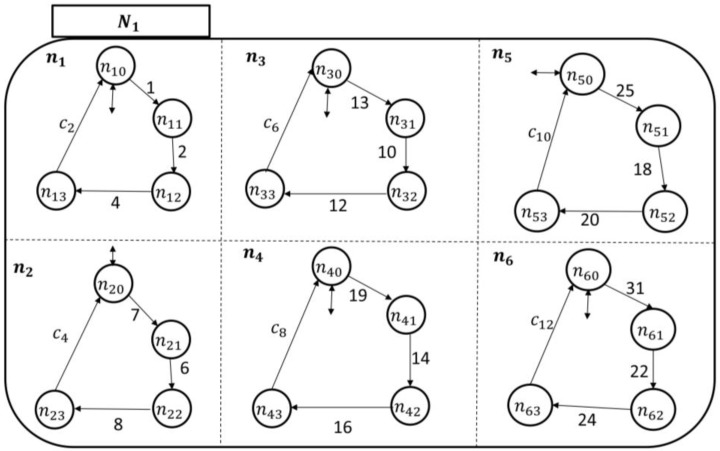
The nursing station automaton $\left(N_{1}\right)$ transitional model.

**Figure 15:**
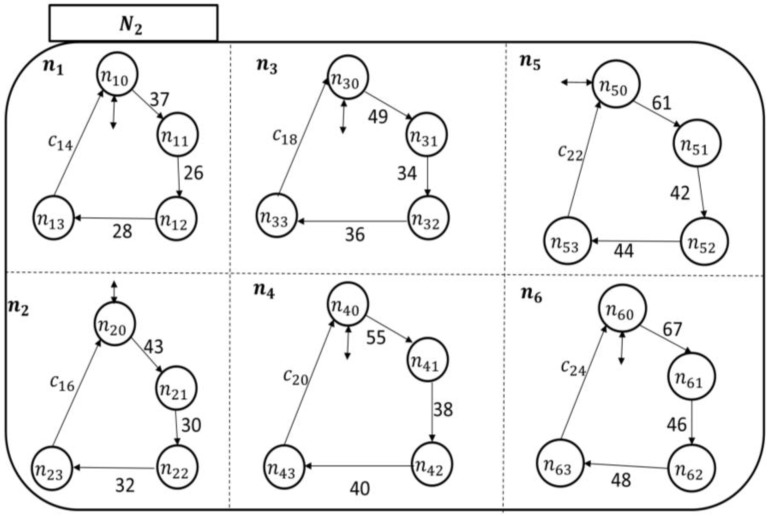
The nursing station automaton $\left(N_{2}\right)$ transitional model.

To monitor the six symptoms of the patients (automata), each nursing station has six holons, as shown by their transitional models in Figure 14 and Figure 15.

**Figure 16:**
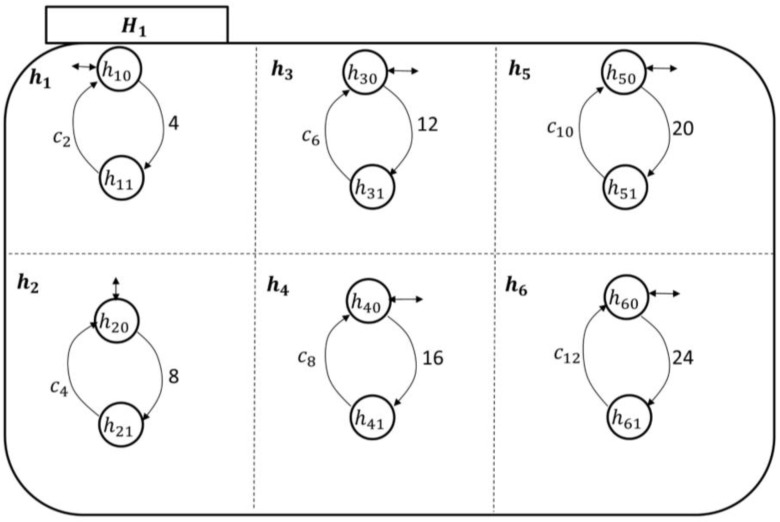
The hospitalist automaton $\left(H_{1}\right)$ transitional model.

**Figure 17:**
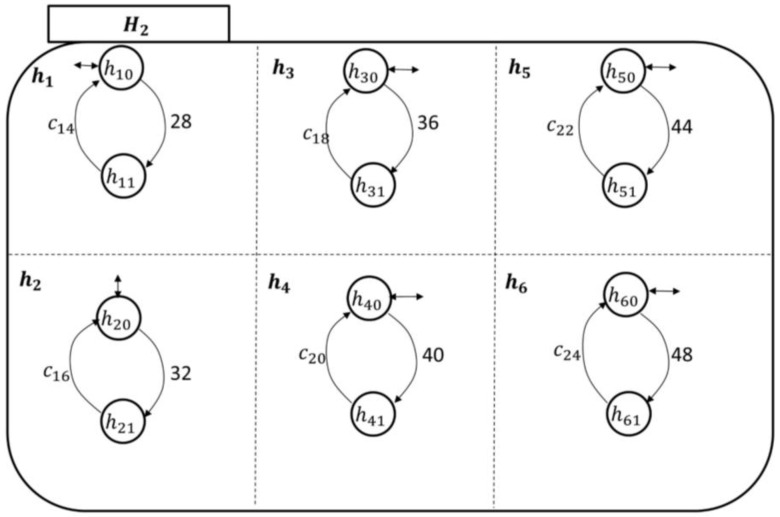
The hospitalist automaton $\left(H_{2}\right)$ transitional model.

The hospitalist automata are shown in Figure 16 and Figure 17. Their enabling events can be listed in the following two sets, respectively, as follows:

(20) $$\Sigma_{{hcp}_{1}}=\left\{c_{2},c_{4},c_{6},c_{8},c_{10},c_{12}\right\}$$

and

(21) $$\Sigma_{{hcp}_{2}}=\left\{c_{14},c_{16},c_{18},c_{20},c_{22},c_{24}\right\}.$$

Clearly, as demonstrated in the transitional models, the alphabet of transition labels $\Sigma =\Sigma_{{pt}_{1}}\dot{\cup }\;\Sigma_{{pt}_{2}}\dot{\cup }\;\Sigma_{{ncp}_{1}}\;\dot{\cup }\;\Sigma_{{ncp}_{2}}\;\dot{\cup }\;\Sigma_{{hcp}_{1}}\;\dot{\cup }\;\Sigma_{{hcp}_{2}}$. The simple/local states in each holon are as discussed in Section 3. For instance, if $s_{10}$ is among the current local states of the patient automaton $PA_{1o}$, then the patient is not experiencing pain currently (*idle/initial state*). Notice that in both monitoring agents, namely $G_{1}$ (which includes the concurrent automata $PA_{1o},\;N_{1},\;\mathrm{and}\;H_{1}$) and $G_{2}$ (which includes the concurrent automata $PA_{2o},\;N_{2},\;\mathrm{and}\;H_{2}$), the coupling events are incorporated, respectively, by

(22) $$E_{1}=\left\{1,7,13,19,25,31,2,4,6,8,10,12,14,16,18,20,22,24,\;c_{2},c_{4},c_{6},c_{8},c_{10},c_{12}\right\}$$

and

(23) $$\begin{array}{c} E_{2}\\ =\left\{37,43,49,55,61,67,\;26,28,30,32,34,36,38,40,42,44,46,48,c_{14},c_{16},c_{18},c_{20},c_{22},c_{24}\right\}. \end{array}$$

States $n_{j0}$ and $h_{j0}$ in $N_{i}$ and $H_{i}$ respectively represent the initial (and marked) state (i.e., the *idle state*). The rest of the states in such holons represent the process of a symptom investigation by the care provider. After completing an investigation of a symptom using a certain mechanism consistent with care/administrative procedures, the care provider automata return back to the corresponding initial states. We expect these care/administrative procedures to be focused on managing the patient’s symptoms and conditions; they may include accessing patient behavioral information stored on the server to further investigate the case (see the next section for a discussion).

Suppose, after considering each patient’s medical condition, the care provider only wants to react to certain key symptoms of a patient, not all symptoms. For example, the hospitalist might inform the nursing station to contact the patient, modeled by $PA_{1o}$, if he/she experiences pain, bleeding, and/or infection. Thus, to manage the symptom(s) more effectively, the reporter must only report (to $N_{1}$) events 1, 13, and/or 19, which are a subset of the coupling events, when executed by $PA_{1o}$. Assume the events to be watched for regarding $PA_{2o}$ are 37 and/or 61, which represent the “occurrences” of the pain and swelling symptoms.

**Figure 18:**
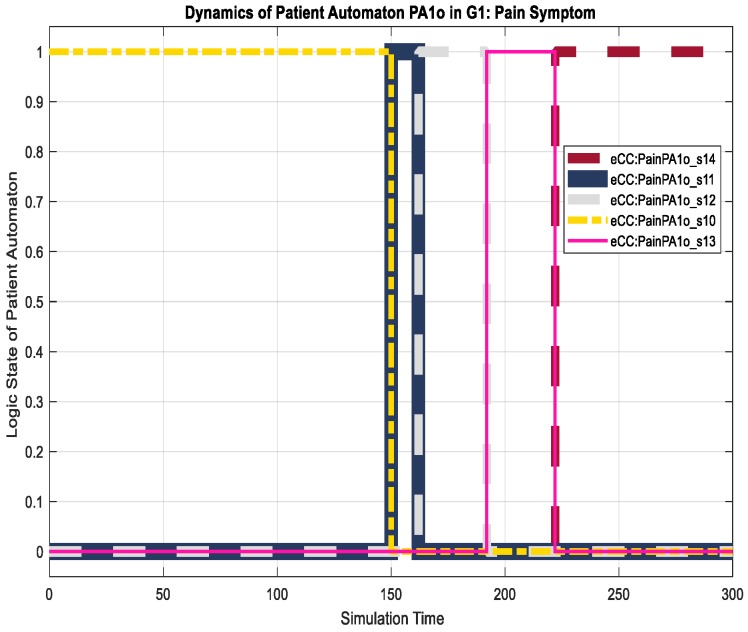
Dynamics of the patient, $PA_{1o}$, in terms of the pain symptom.

**Figure 19:**
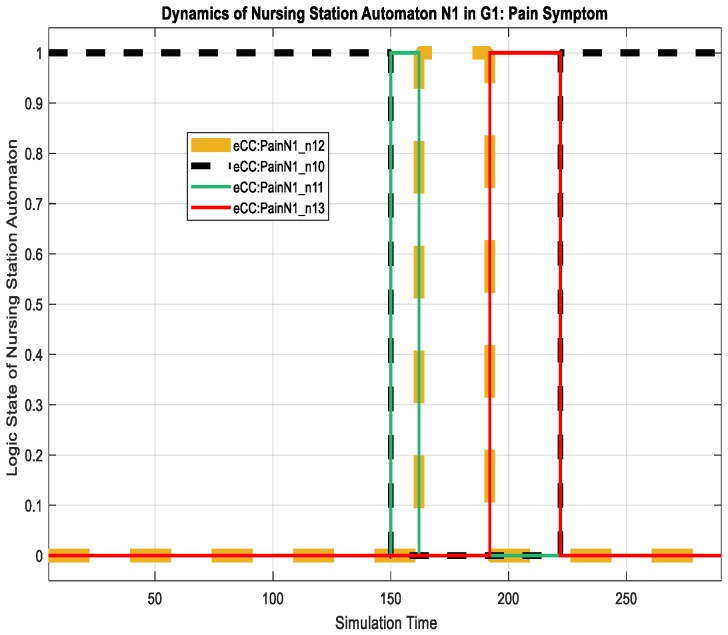
Dynamics of the nursing station, $N_{1}$, in terms of monitoring the pain symptom.

**Figure 20:**
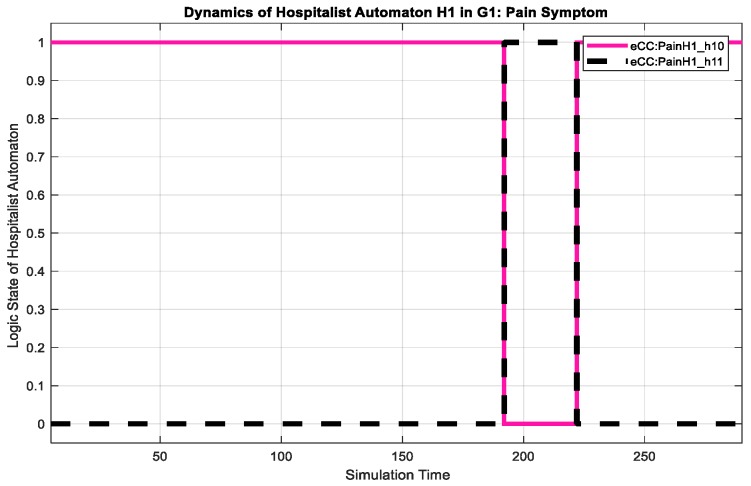
Dynamics of the hospitalist, $H_{1}$, in terms of developing a care plan for the intervention to avoid the pain symptom.

The corresponding eCC system was simulated using Matlab software (version 17 with Simulink/Stateflow). Since the eCC is expected to operate for an extended time, the simulation is a snapshot of the eCC’s operating life. Figure 18, for instance, shows the dynamics of the patient automaton $PA_{1o}$ for the given snapshot in terms of the pain symptoms. The patient starts to experience pain at around 150 min of the simulation time as he/she transitions to state $s_{11}$, shown in Figure 18 as eCC:PainPA1o_s11. Since pain is among the key symptoms to be closely watched, the occurrence of event 1 is communicated with the nursing station causing it to transition to state $n_{11}$ (i.e., eCC:PainN1_n11), and an investigation process is started at around 160 min, as depicted in Figure 18 and Figure 19. Note that any current state of the automata is given a binary value of 1. As the investigation of the pain symptom progresses by $N_{1}$ and $H_{1}$, the patient automaton is updated to reflect the progression. For example, when hospitalist $H_{1}$ reviews/develops/approves the “intervention,”, represented by a transition from the state $h_{11}$ (eCC:PainH1_h11) back to the state $h_{10}$ (eCC:PainH1_h10), the patient and the nursing station are notified at a simulation time of approximately 225 min (refer to Figure 18, Figure 19 and Figure 20). The patient is not able to stop this process; it only stops or is finalized formally by the care provider. These behaviors indicate the essential integration needed between the patient;s engagement and the care provider’s administrative role for the eCC to be effective. In other words, patients must comply and report any observed symptoms. Once reported, the corresponding care provider will be at a state of investigation until the “intervention” decision has been made and the “file” is closed. Such integration is conducted in an interactive way between the automata, which also can be shown by way of simulation, as follows.

**Figure 21:**
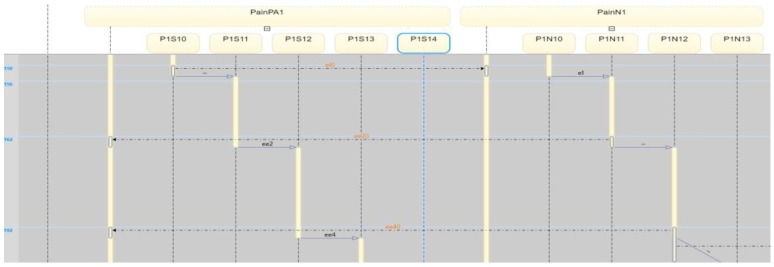
The $PA_{1o}$ automaton interacts with the nursing station $\left(N_{1}\right)$ regarding the pain symptom via time-stamped coupling events.

**Figure 22:**
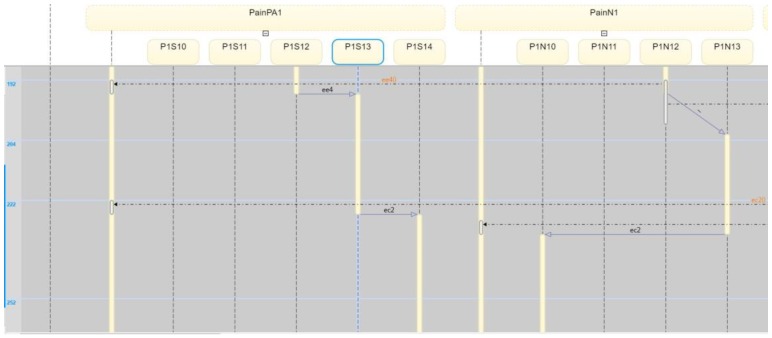
The $PA_{1o}$ automaton interacts with the care provider regarding the pain symptom via time-stamped coupling events. This view also shows the hospitalist behavior, namely, the coupling event $c_{2}$ (or ec2).

Interactions between the patient automaton and the care provider automata are exercised via coupling/shared events. We used the Sequence View Tool in Matlab to show such interactions in this hierarchal modeled system. As shown in Figure 21, the patient $PA_{1o}$ experiences some pain, and hence, starts his/her engagement by making an internal transition in the pain holon in $PA_{1o}$ from $s_{10}$ to $s_{11}$ (represented in Figure 21 by P1S10 and P1S11, respectively). Event 1 (shown in Figure 21 as e1()) is communicated, as characterized by the dashed black line, to the pain-investigation holon in $N_{1}$ (shown in Figure 21 as PainN1). The system state changes from “no pain” in $PA_{1o}$ to “pain,” which requires the care provider to react since it is a key symptom/state. When the investigation process of the pain symptom is started by $N_{1}$, an internal transition takes place from state $n_{11}$ to $n_{12}$ (shown in Figure 21 as P1N10 and P1N11, respectively), and the associated event, namely 2 (or ee2()), is communicated to the patient, causing a transition from $s_{11}$ to $s_{12}$ (P1S10 and P1S11, respectively), effectively showing the transparency of the process and the administrative role of the care provider facilitated by the eCC system. The transition dynamics of the hospitalist as he/she is fulfilling her/his administrative role are also shown in Figure 22. When the intervention plan to avoid/manage the pain symptom has been approved, a corresponding event, namely, $c_{2}$ (ec2), is generated by the hospitalist automaton as shown in Figure 22. This causes the nursing automaton and the patient automaton to transition to state $n_{10}$ (or P1N10 in Figure 22) and $s_{14}$ (or P1S14 in Figure 22), respectively. One can follow the dynamics and ongoing interactions via the time-stamped coupling events and the corresponding transitions in $PA_{1o}$, $N_{1}$**,** and $H_{1}$ holons, using Figure 18 through to Figure 22.

**Figure 23:**
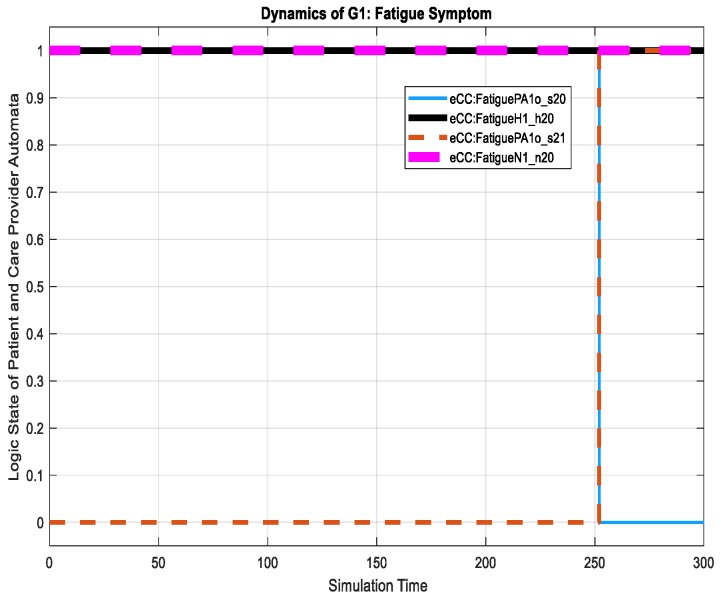
Dynamics of $G_{1}$ in terms of the fatigue symptom.

**Figure 24:**
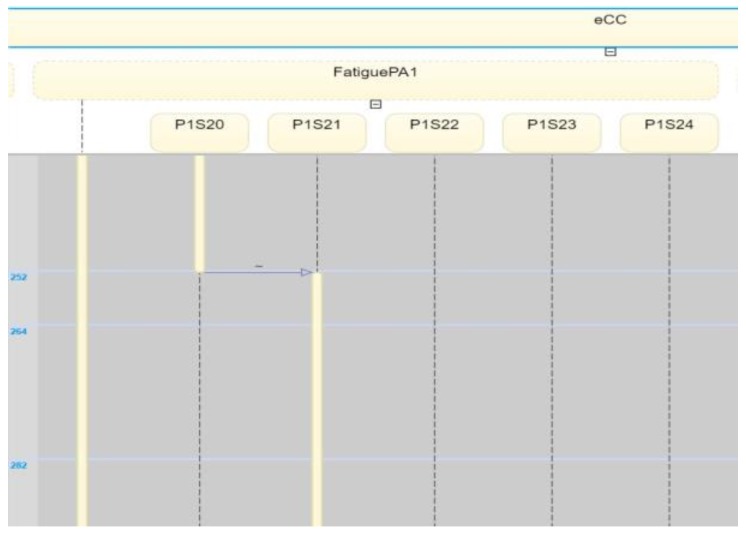
Internal transition of $PA_{1o}$ in holon $s_{2}$ from $s_{20}$ to $s_{21}$ (P1S20 to P1S21); this event was not shared with the nursing station.

An example of a non-key symptom is shown in Figure 23, where the patient starts to feel fatigue, shown by a transition in the $s_{2}$ holon from state $s_{10}$ to state $s_{21}$ (on the figure shown as eCC:FatiguePA1o_s21). For this transition, notice that the care provider dynamics remain unchanged. They maintain their *idle states* at $n_{20}$ and $h_{20}$ in $N_{1}$ and $H_{1}$, respectively, regarding the fatigue symptom investigation. The two *idle states* are shown in Figure 24 by eCC:FatigueN1_n20 and eCC:FatigueH1_h20. Coupling event 7 is not communicated to the care provider (i.e., to simulate events-erasing functionality by the reporter), as shown in Figure 24, indicating an efficient administrative role for the care provider and unrestricted engagement of the patient made possible by the eCC system.

**Figure 25:**
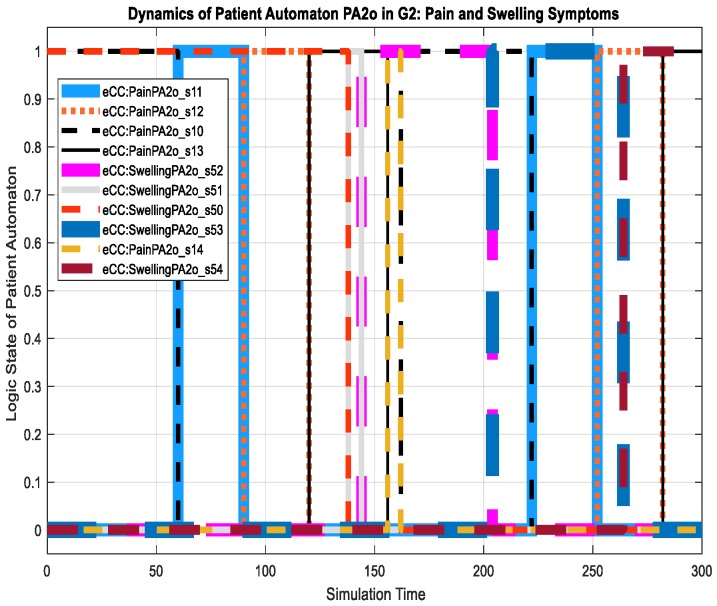
Dynamics of patient automaton $PA_{2o}$ in $G_{2}$ in terms of the key symptoms: pain and swelling.

**Figure 26:**
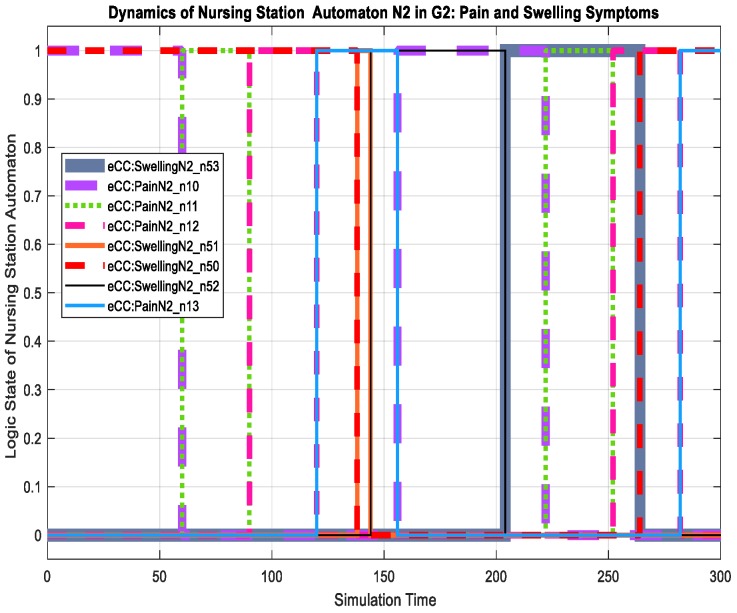
Dynamics of patient automaton $N_{2}$ in $G_{2}$ in terms of monitoring the pain and swelling symptoms.

**Figure 27:**
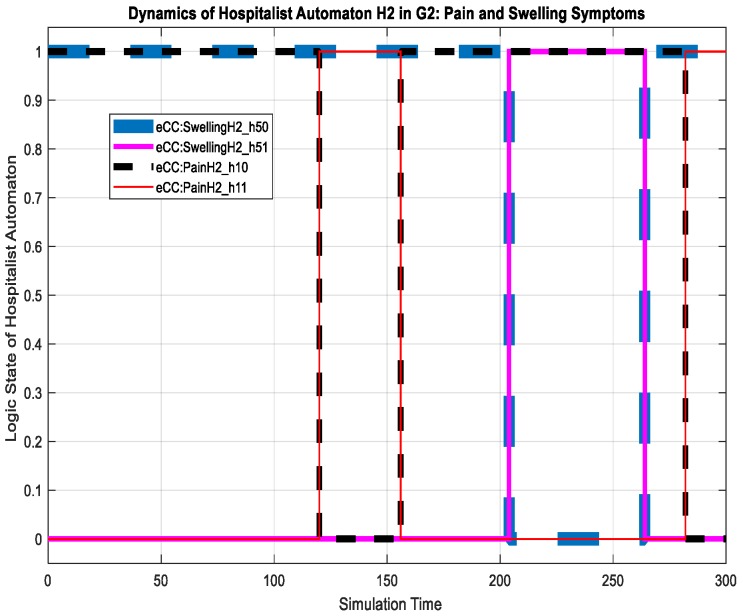
Dynamics of patient automaton $H_{2}$ in $G_{2}$ in terms of monitoring the pain and swelling symptoms.

For the patient automaton $PA_{2o}$, we discuss its dynamics in terms of the key symptoms only. The patient experienced the pain (transition to state eCC:PainPA2o_s11) and swelling (transition to state eCC:SwellingPA2o_s51) symptoms at simulation times of approximately 70 and 138 min, as shown in Figure 25. Both symptoms are monitored by the care provider. The nursing station thereby reacts to the pain symptoms by transitioning to eCC:PainN2_n11 and eCC:SwellingN2_n51, respectively, as shown in Figure 26. Meanwhile, the patient automaton $PA_{2o}$ is also notified of the initiation of symptom investigation via transitions to eCC:PainPA2o_s12 and eCC:SwellingPA2o_s52. The remainder of the relevant dynamics, including those of the hospitalist, can be followed using Figure 25, Figure 26 and Figure 27. For example, the hospitalist approves care plans for both the pain and swelling symptoms at around 155 and 265 min of the simulation time, respectively, shown by the transitions to states $h_{10}$ and $h_{50}$ (eCC:PainH2_h10 and eCC:SwellingH2_h50, respectively), in Figure 27. The nursing station, as expected, transitions to $n_{10}$ and $n_{50}$ (eCC:PainN2_n10 and eCC:SwellingN2_n50) in Figure 26. The approval of the care plans also causes the patient automaton to transition to states $s_{14}$ (eCC:PainPA2o_s14) and $s_{54}$ (eCC:SwellingPA2o_s54), in Figure 25. Notice that by comparing Figure 19, Figure 20, Figure 26 and Figure 27, one can see that the eCC allows the care provider to handle patients independently and simultaneously, mimicking the behavior of handling patients in HCOs.

The simulation results demonstrate the *patient-centric approach* adopted in this study for real-time patient monitoring. The focus of the system is *centered* on and is *customized* to the patient at all times. The patient is able to report a symptom from the home/work place, and can receive updates on the symptoms’ investigations. It has been shown that each patient can report more than one symptom simultaneously and receive updates about each one independently. Furthermore, the patient’s engagement with his/her own care is facilitated by treating each patient as a generator/automaton that can initiate/generate events. These, among other functionalities, show that the eCC system effectively enables a collection of services including (1) “optimal” real-time interactions between the patient and care provider to automate identifying patients with higher risk of readmission and observance of care plans/investigation developments; (2) the ability to include a higher authority to automate the supervisory function; (3) the continuity of care during TCPs; (4) the ability to independently maintain key symptoms/information customized to each patient and his/her health developments; (5) the possibility of timely proactive planning, intervention, and coordination of services (e.g., contacting the patient, scheduling a home visit, etc.) based on the patient’s needs; and (6) electronically documenting and storing information regarding patients’ health-related activities and conditions on a daily basis during TCPs. These services of extended care permit [9,28] effective and efficient support and sustainable discharge which are vital for reducing unplanned readmission rates and in return, improve the QOC and reduce COC [6,7].

While other approaches to monitoring patients outside formal HCOs are found in the existing literature (see, for example references [10,26,27,28,36]), they are observed (in light of their functionalities) to lack the needed services of extended care and sustainable discharge. In the systems of references [10,26,27,28], patients are required to periodically enter information related to their conditions. This information becomes available to prescribed care providers to look at and assess. These systems may be equipped with health analytics tools. However, they lack automated processes to point out patients who need immediate help/intervention in real-time. They require care providers to review the data of all patients to sort out the ones with higher risk of readmission, and then intervene. This process of vetting a large number of patients not only is extremely cumbersome, but also, without being automated, cannot be guaranteed to take place in a timely manner. The system proposed by reference [36] requires special medical devices to monitor patients remotely. It collects data about patients’ health and health-related conditions, and then, uses a rules-based approach so that the system can operate at a logical level and issues warnings/alerts to caregivers. The system is designed to be more appropriate for interactions between a patient and his/her family members (caregivers). Hence, it is inadequate for use in handling issues like unplanned hospital readmissions. This is due to the following reasons: the system is “rigid” and it lacks the ability to adjust in real-time to account for patients’ health developments (improving or worsening conditions) during TCPs. Furthermore, the system does not fully observe the uniqueness of each patient. Moreover, none the reviewed systems include the capability of automating information flow from care providers back to patients which may degrade the extended care required for patient discharge sustainability. A further discussion on similar existing systems and their limitations can be found in reference [7].

To this end, and for our purposes, the eCC system can be an addition or a substitute to these systems as well as all the following inefficient/ineffective current HCOs practices [7]:

(1) A health professional calling all discharged patients, without distinguishing those who actually need help and the time in which they need it.
(2) Automated calls with no health professional in the loop which can be difficult to personalize.
(3) Unnecessary follow-up home visits by health professionals.
(4) Extending unnecessary patient hospital stays which could delay serving other patients.

The eCC functionalities are conducted mainly by two healthcare software applications, namely, an initiator and an inquirer, which are expected to behave as shown in the simulation above when developed using the discussed models in Section 3. The following section discusses a more detailed plan of system implementation and use.

## 5. High-Level Conceptual Design and Basic Plan for System Implementation

**Figure 28:**
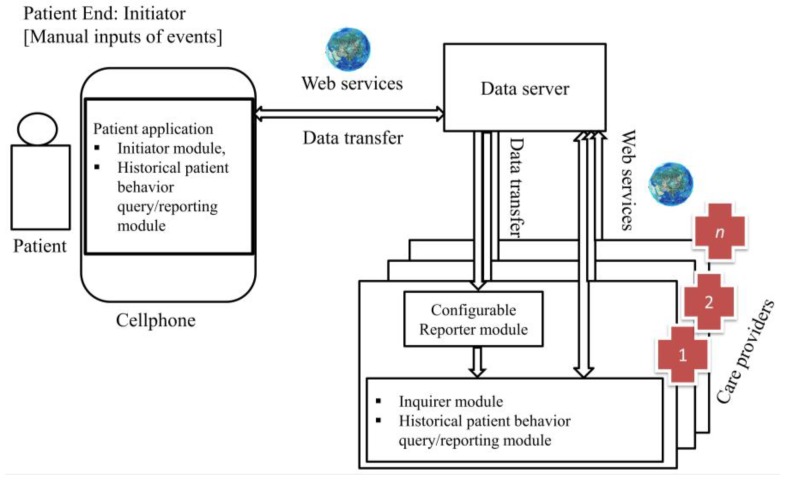
Conceptual design of the eCC system: patients connected to care providers [7].

Developing an implementation plan for such a platform would be a considerable separate project all on its own. Herein, we provide a high-level discussion on developing the proposed eCC platform. The eCC platform connects patients (initiators) to care providers (inquirers); the system can be decomposed into primary components (Figure 28), as follows:

(1) A patient-end, *the initiator*, which interacts with the system through a personal mobile device software application, denoted the *initiator software application* (PIapp). The PIapp consists of two primary modules: (i) the initiator module; and (ii) the historical patient behavior query/reporting module.
(2) A care provider-based receiving end, denoted *the inquirer*, which has a reporter. The inquirer’s interaction is through a receiving-end software application, denoted the *inquirer software application* (CIapp), downloaded to the healthcare organization’s computing devices from the server. The CIapp is developed in accordance with the care-provider functionality and reporter models, as discussed previously. The CIapp consists of three primary modules: (i) the configurable reporter module; (ii) the inquirer module; and (iii) the historical patient behavior query/reporting module.

The initiator module was developed on the basis of the universal patient model (UPM). The model includes discrete patient states (nearly all possible symptoms and health-related activities) and the corresponding state-associated transitions. In the initiator module, the patient can operate only between the transitions; in other words, he/she can enable an event manually by clicking a button. An icon-based interfacing app is ideal for this type of application, especially as the model will be represented by a transition graph of a finite-state automaton. Events associated with specific symptoms remain disabled until they are enabled by the patient. Once an event is enabled, a transition occurs. Every transition takes the patient from one state to another (e.g., a transition may take a patient from the “no pain” state to the “experiencing pain” state), and the patient remains in a particular state until an associated event occurs. The patient’s smartphone sends the most recent event (data) to the server via a web service. The server documents the event and a copy passes in real-time to the corresponding care providers’ computing devices where the CIapp is installed. We would like to acknowledge potential complexities here that will need to be defined and tackled. For example, an event could be generated by the initiator by mistake. One would need to include a *layer of confirmation* before event generation at the initiator end to allow for fewer or no mistakes in event generation. This could also be applied at the inquirer end so that activated actions are highly accurate.

The web services are responsible for establishing communication securely between the server platform and the mobile applications using end-to-end encryption over the internet. We expect the server (cloud-based or dedicated) to be hosted by a private or governmental body, and to provide services and support for targeted patients and healthcare organizations (at the local, provincial, or national level). Instead of a private or governmental body, a single or group of healthcare organizations may support their own server as part of post-discharge support and continuity care services for their patients. Along with data-processing, computing power, and storage capacity, the server provides the necessary functionality for users and the communication of services (e.g., role management).

The primary users include patients in the transitional care period, nurses at nursing stations in hospitals, and hospitalists. At the time of hospital discharge, the care provider, through the CIapp, initiates a time-limited connection with the patient using the patient’s phone number as his/her identifier. In this manner, the two ends of the eCC are now connected, and any qualified (or prescribed) event occurring at the patient’s end will affect the CIapp, as explained above. The duration of transitional care support must also be defined at this time. As explained above, there are primarily two different choices based on the patient’s attributes and medical condition. Upon completion of the transitional care period, the patient may be disconnected automatically.

The PIapp, which can be downloaded to the patient’s smartphone from the server, executes major components of eCC functionality: generating events, data transfer, etc. The same is true of the CIapp, which filters out all of the non-coupling, non-observable events via the configurable reporter module; generates the corresponding events when any of the coupling and observable events occur, as per the inquirer module; and accesses historical data to facilitate patient evaluation, if necessary. To help minimize hardware cost, we expect that the most common mobile operating systems (e.g., Android, iOS, and Windows) will be supported, making use of existing equipment.

## 6. Conclusions

Unplanned hospital readmissions bring large social and economic costs to both patients and healthcare organizations. We proposed a mobile health platform designed to reduce unplanned readmissions, thereby reducing costs while maintaining or improving the overall quality of care. We configured the platform to mimic intelligent behavior in hospitals—in particular, the behavior of delivering timely “care” to patients based on their needs. This requires acting (in real-time) on patients’ symptoms which are considered part of the system state variables to which the care provider automata react. Since there are many symptoms, the eCC system has been provided with the capability to be customized to each patient’s key symptoms independently. The system can be reconfigured in real-time based on patient health developments, hence fully realizing patients’ uniqueness.

It has been shown that discharged patients and involved care providers can together be treated as a discrete event system (DES). Such treatment allows for a systemic design approach to a system and facilitates communications for e-healthcare services provision. The system can effectively and efficiently convey symptom information and symptom investigation processes between patients and care providers. The proposed system is generic; thus, in principal, it can be used by any hospital to monitor targeted symptoms of any patient. An illustrative simulation was provided to help understand the workings of the system, in which the roles of patients and care providers are vital. We also provided a high-level discussion of a basic implementation plan. The UPM and care provider models are the basis on which to develop the main two components of the eCC, specifically, the initiator and the inquirer. Some potential complexities were identified to be tackled at the time of implementation.

An extension to this work could include linking the proposed system to biomedical devices and sensors in a way that automates the generation of symptom-related events as they occur. Hence, as an automaton, when the patient experiences different symptoms, she/he transitions between states that represent those symptoms automatically. A detailed study of the social and economic benefits of the proposed system would be an important integral part of the system implementation.

## Appendix A

**Table A1:** Symptoms’ superstates.

The Symptom Definition	The Symptom Superstate Symbol
pain	$s_{1}$
fatigue	$s_{2}$
headache	$s_{3}$
infection	$s_{4}$
swelling	$s_{5}$
fever	$s_{6}$
weight loss	$s_{7}$
nausea	$s_{8}$
blood sugar level	$s_{9}$
sore throat	$s_{10}$
runny or stuffy nose	$s_{11}$
vomiting	$s_{12}$
stress	$s_{13}$
shortness of breath	$s_{14}$
diarrhea	$s_{15}$
depression	$s_{16}$
anxiety	$s_{17}$
constipation	$s_{18}$
cough	$s_{19}$
bleeding	$s_{20}$
itching	$s_{21}$
skin rash	$s_{22}$
dizziness	$s_{23}$
confusion	$s_{24}$
irritability	$s_{25}$
asthma	$s_{26}$
hypoesthesia (numbness): partial loss of tactile sensation	$s_{27}$
skin ulcer	$s_{28}$
pustule	$s_{29}$
anorexia	$s_{30}$
hearing loss	$s_{31}$
hypertension	$s_{32}$
tinnitus	$s_{33}$
anaphylaxis	$s_{34}$
paresthesia: a sensation of burning, prickling, itching, or tingling of the skin	$s_{35}$
bloating	$s_{36}$
cramp	$s_{37}$
epileptic seizure	$s_{38}$

Each one of these superstates has components.

## Appendix B

To systematically compute a number by which an event in a holon can be labeled, we propose the following algorithm, assuming identical holon structure (except for the transition labeling), like we presented earlier in Figure 7, Figure 9 and Figure 11.

First, note that all events appearing in $N_{i}$, and $H_{i}$ are found in their counterpart $PA_{io};$ thus, our computation is limited to finding the labeling number of events for $PA_{io}$. For $\sigma ∊\Sigma_{pt_{j}}$ in $s_{j}$ $s_{j}∊{ℰ}^{+}\left(PA_{io}\right)$ (see reference [32] for details about ${ℰ}^{+}$). First, compute the labeling number for events appearing in $PA_{1o}$ by

(A1) $$y_{n}=2q_{1}\left(j,z,n\right)+1,$$

where n=1, 2, or 3. In the holon $s_{j}$:

(1) $y_{1}$ is the labeling number of the event where the transition exits $s_{j0}$ and enters $s_{j1}$,
(2) $y_{2}$ is the labeling number of the event where the transition exits $s_{j1}$ and enters $s_{j0}$, and
(3) $y_{3}$ is the labeling number of the event where the transition exits $s_{j4}$ and enters $s_{j0}$.

The parameter z is equal to the number of $\sigma ∊\Sigma_{pt}$ that appear in the holon $s_{j}$, which is 3 here, and

$$q_{1}\left(j,z,n\right)=z\left(j-1\right)+\left(n-1\right).$$

Now, for $\sigma ∊\Sigma_{npt_{j}}$, use the following equation:

(A2) $$e_{f}=2\;q_{2}\left(j,f,w\right)+2,$$

where f=1 or 2. In the holon $s_{j}$,

(1) $e_{1}$ is the even labeling number of the event where the transition exits $s_{j1}$ and enters $s_{j2}$, and
(2) $e_{2}$ is the even labeling number of the event where the transition exits $s_{j2}$ and enters $s_{j3}$.

The parameter w(=2) is the number of $\sigma ∊\Sigma_{npt}$ that appear in the holon $s_{j}$, and

$$q_{2}\left(j,f,w\right)=w\left(j-1\right)+\left(f-1\right).$$

For $\sigma ∊\Sigma_{hpt_{j}}$, we know that $c_{p}$ labels a transition leaving $s_{j3}$ going to $s_{j4}$ in the holon $s_{j}$; thus, the following mathematical relation can be used,

(A3) p=2j.

For all other $PA_{io}$ where i>1, the above equations are modified as follows

(A4) $$\begin{array}{c} y_{n}=2q_{3}\left(b,z,n\right)+1\\ q_{3}\left(b,z,n\right)=z\left(b-1\right)+\left(n-1\right)\\ e_{f}=2\;q_{4}\left(b,f,w\right)+2\\ q_{4}\left(b,f,w\right)=w\left(b-1\right)+\left(f-1\right)\\ p=2b \end{array}$$

where $b=\Sigma_{i=1}^{i-1}g_{i}+j$ and $g_{i}$ is the number of holons in $PA_{io}$; in the example above, there are six holons.
